# Social induction and the developmental trajectory of participation in intergroup conflict by vervet monkeys

**DOI:** 10.1017/ehs.2025.7

**Published:** 2025-03-13

**Authors:** Madison Clarke, Tyler Bonnell, Rosemary Blersch, Christina Nord, Chloé Vilette, Christopher Young, Peter Henzi, Louise Barrett

**Affiliations:** 1 Department of Psychology, University of Lethbridge, Lethbridge, AB, Canada; 2 Applied Behavioural Ecology and Ecosystems Research Unit, University of South Africa, Pretoria, South Africa; 3 Department of Computer Science, University of Calgary, Calgary, AB, Canada; 4 Neuroscience and Behavior Unit, California National Primate Research Center, Davis, CA, USA; 5 Department of Psychology, Nottingham Trent University, Nottingham, UK

**Keywords:** collective action, cooperation, aggression, non-adults, development, social networks

## Abstract

We assess the proposition that intergroup conflict (IGC) in non-human primates offers a useful comparison for studies of human IGC and its links to parochial altruism and prosociality. That is, for non-linguistic animals, social network integration and maternal influence promote juvenile engagement in IGC and can serve as the initial grounding for sociocultural processes that drive human cooperation. Using longitudinal data from three cohorts of non-adult vervet monkeys (*Chlorocebus pygerythrus*), we show that non-adults are sensitive to personal (age) and situational risk (participant numbers). The frequency and intensity of participation, although modulated by rank and temperament, both mirrors maternal participation and reflects non-adult centrality in the grooming network. The possibility of social induction is corroborated by the distribution of grooming during IGC, with non-adults being more likely to be groomed if they were female, higher-ranking and participants themselves. Mothers were more likely to groom younger offspring participants of either sex, whereas other adults targeted higher-ranking female participants. Although we caution against a facile alignment of these outcomes to human culturally mediated induction, there is merit in considering how the embodied act of participation and the resultant social give-and-take might serve as the basis for a unified comparative investigation of prosociality.

## Graphical abstract

**Figure:**
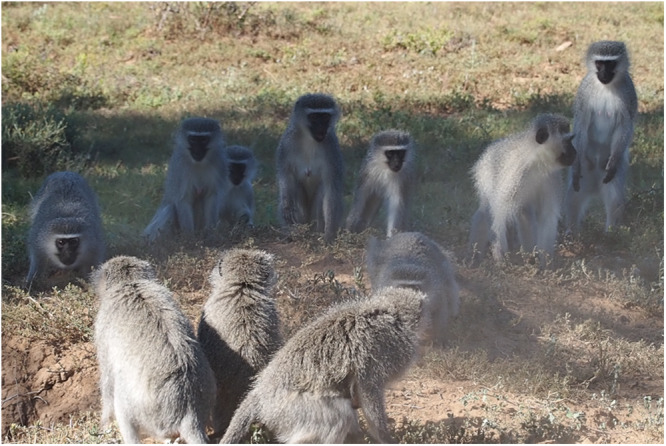

## Social media summary

Social induction and the developmental trajectory of participation in intergroup conflict by vervet monkeys.

## 1. Introduction

Human social groups are characterized by the sheer scale and diversity of the cooperative interactions of their members (Boyd & Richerson, 2009), which can extend well beyond ties of kinship and incorporate extreme altruism (Henrich & Henrich, 2006; Rand & Nowak, 2013). Choi and Bowles (2008; see also Bowles, 2007), among others, have argued that human hyper-cooperation is essentially parochial, being restricted primarily to members of the same group and coupled to the distinctively non-cooperative violent conflict, principally warfare, that has historically characterized the interactions between groups.

It is the deep hominin roots of this coevolutionary linkage of in-group altruism and out-group aggression (Bowles, 2009) that links human cooperation to its equivalents in other obligate social species, most notably anthropoid primates (Crofoot & Wrangham, 2010). Intergroup conflict (IGC) across a wide range of such species generally involves two or more group members, is also aggressive, and can readily escalate into violent or lethal physical attack (Cords, 2002; Crockett & Pope, 1988; Harrison, 1983; Hausfater, 1972; Miller, 1998; Palombit, 1993; Wilson et al., 2014), with all the attendant risks that this carries for participants.

Where such goal-directed collective action constitutes a public good, such that benefits but not costs are shared by non-participants, one central question concerns the mechanisms that promote participation and the prevention of exploitation by ‘free-riders’ (Willems, Hellriegel, & van Schaik, 2013). Proximally, there are regulatory processes directed at rewarding participants and punishing defectors that, at least in broad relief, are shared by humans and non-humans, and which serve directly to promote the production of a public good (Arseneau-Robar et al., 2016; Bshary, Richter, & van Schaik, 2022; Cheney & Seyfarth, 1987; Gao, Wang, Pansini, Li, & Wang, 2015; Glowacki & Wrangham, 2013; Kowalewski & Garber, 2015; Mathew & Boyd, 2011; Raihani, Thornton, & Bshary, 2012). More distally, however, human parochial altruism is buttressed by the developmental inculcation of prosocial norms that are fundamentally and distinctively rooted in cultural learning, norm psychology and the transformative effects of language (Chudek & Henrich, 2011; Richerson & Boyd, 2005; Smith, 2010).

Although human infants have a biological predisposition to altruistic behaviour, not apparent in chimpanzees (*Pan troglodytes*, Warneken & Tomasello, 2009), they are also born into culture and internalize cultural ‘rules’ through the necessary mediation of enculturated others and their participation in cooperative cultural activity (Moll & Tomasello, 2007; Vygotsky, 1987). The consequences of these features of human life are that inequity aversion and altruistic sharing (egalitarianism) emerge prior to puberty and in lockstep with parochialism (Fehr, Bernhard, & Rockenbach, 2008), constituting a fundamentally powerful force in overcoming the undermining of collective action by the defection of ‘rational egotists’ (Ostrom, 2000), and thereby providing a mechanistic basis for Bowles’ arguments (Bowles, 2008).

Given good evidence for variability within and across individuals in participation during IGCs (Cords, 2007; Crofoot & Gilby, 2012; Kitchen & Beehner, 2007; Kowalewski & Garber, 2015), the comparative question that we wish to address is whether, in the absence of the powerful cultural forces available to humans, there are developmental trajectories in non-human animals that might underpin differential and variable participation. In the absence of both a cultural toolkit and a capacity for empathy (Vasconcelos, Hollis, Nowbahari, & Kacelnik, 2012), the principal possibility that suggests itself is that participation in IGCs promoted through social network structure (Crofoot, Rubenstein, Maiya, & Berger-Wolf, 2011; Glowacki et al., 2016; Siegel, 2009) might serve as an analogue for the emergence of baseline prosociality in non-human societies.

At the same time, despite the potential severity of the consequences of physical aggression with adults (Silk, Samuels, & Rodman, 1981), and the theoretical expectation that juveniles should be risk-averse (Janson, 1993), there is evidence that juvenile blue monkeys (*Cercopithecus mitis*), for example, participate in aggressive IGCs at rates that increase with age (Cords, 2007). This phased increase has also been observed in lions (*Panthera leo*), where the involvement of juvenile females is tied to the numbers of both defendants and intruders, and indicates the early development of an appropriate sensitivity to context and risk (Heinsohn, Packer, & Pusey, 1996). These examples make it clear that physical immaturity need not impede participation in risky ventures and confirm that a developmental approach to understanding the patterns of adult intergroup behaviour is likely to pay dividends.

Vervet monkeys (*Chlorocebus pygerythrus*) provide an excellent opportunity to investigate these issues. They are a widely distributed African group-living Old World monkey species with female philopatry and, like the closely related blue monkeys, have long been known for aggressive IGCs (Cheney, 1981; Harrison, 1983; Struhsaker, 1967) in the context of resource defence, and typified by considerable variation in adult participation that is tied to differential costs and benefits (Arseneau-Robar et al., 2016). Here we take advantage of longitudinal developmental data from three birth cohorts of vervet monkeys to consider four questions.

Principally, we wish to consider the proposition that the extent of social integration is positively linked to cooperation during IGC. In humans, altruistic cooperation is more likely where individuals are both strongly connected to a few other individuals but where such clusters are also connected to one another by bridging individuals (Burt, 2000). Juvenile vervets form distinctive and stable ego-network structures (Vilette, Bonnell, Dostie, Henzi, & Barrett, 2022) that, although they do not mirror maternal networks (Jarrett, Bonnell, Young, Barrett, & Henzi, 2018), nevertheless have maternal bonds at their core (Vilette, Bonnell, Dostie, Henzi, & Barrett, 2023). Consequently, we use the extent of maternal participation in IGC, alongside a juvenile’s eigenvector centrality (EC), which reflects the depths of the latter’s penetration in the relevant network (Brush, Krakauer, & Flack, 2013), to predict participation rates.

Second, we expect juvenile participation to be modulated by intrinsic individual attributes such as sex, dominance rank and neophilia. We cannot specify a directional prediction *a priori* with respect to which sex is more likely to participate, as we have good reasons to predict participation in both sexes: juvenile males, because they are larger than their female age mates (Jarrett et al., 2020), and juvenile females, because they are philopatric (Cords, 2007; Heinsohn et al., 1996). Nevertheless, any differential outcome or encouragement may help clarify underlying processes or selection pressures.

Rank is important because, whether or not IGC delivers a public good, it is reasonable to expect a correlation between the extent of participation and the likelihood of the direct benefits afforded by high rank (Willems & van Schaik, 2015) that are evident in our population (Blersch et al., 2023). We therefore predict that higher-ranking juveniles will participate more frequently than will lower-ranking ones (Cheney, 1981). By the same token, higher-ranking adult female blue monkeys, who do not benefit disproportionately from resource defence, are also more likely to participate in IGC (Cords, 2007). This raises the possibility that the underlying driver actually reflects differences in personality traits. We therefore predict that higher neophilia scores (indicative of greater boldness/exploratory tendencies; Blaszczyk, 2017) will underpin an increased likelihood of participation.

Third, in accordance with the perceived need to balance costs and manage risk, we expect participating non-adults to scale their involvement over time. Principally, we expect older, and therefore larger and more experienced, non-adults to participate more frequently and with greater intensity. Here again, however, involvement may be modulated by rank and neophilia, as well as by external considerations, such as the extent of concurrent participation by others. Both an increase in the overall numbers of participants from their own group (hereafter the focal group) or a numerical advantage over the opposing group may reduce exposure to risk and encourage non-adult participation.

Finally, we expect that although non-adults may not be punished for not participating in IGC, grooming will be used as an incentive to participate (Arseneau-Robar et al., 2016), especially by mothers (Vilette et al., 2023). We therefore predict a positive relationship between non-adults participating and being groomed during the IGC. As grooming may simultaneously encourage future collective action, we also consider whether grooming is preferentially directed at females, who will remain in the group for life, and to younger participants.

## 2. Methods

### 2.1. Study species and population

Data were collected from three adjacent groups (RST, RBM, and PT; Supplementary Table 1) of habituated vervet monkeys with overlapping home ranges on the Samara Private Game Reserve in South Africa (Pasternak et al., 2013). RST and RBM have been studied continuously since 2009. The third group (PT) was added in 2012. All individuals were identifiable using unique facial and body markings. Each group was followed by at least one observer for 10 hours each day, 5 days per week across the study period.

Vervets are seasonal breeders and this, at our study site, results in distinct juvenile cohorts (Blersch et al., 2023). Here we use data from the 2013–2015 cohorts (*N* = 68 infants and juveniles, hereafter non-adults) and followed them until the end of the study in 2018. The subjects ranged in age from 1 day to ∼5 years, with females reaching sexual maturity at between 3.5 and 4.5 years, and males likely to leave their natal groups at ∼4.5 years of age (Henzi et al., 2023).

### 2.2 Data collection

*i. General methods*. We used scan sampling (Altmann, 1974) to collect behavioural and activity data. To do so, we recorded the IDs of all visible individuals, their activity (foraging, resting, grooming, moving, playing), their social partners, and their nearest neighbours during a 10-min window every 30 min (*N* = 754,641 individual data points from 2014 to 2018).

*ii. Social networks*. We used the scan data to generate annual grooming and spatial association networks with the ‘igraph’ package (Csardi & Nepusz, 2006) in R 4.4.2 (R-Core-Team, 2022). As juveniles in our population maintain stable networks with few strong ties and several weak ties regardless of sex (Vilette et al., 2022), we created troop-level networks for each year in the study period. We extracted all occurrences of non-adults being groomed by, or grooming, another individual to generate directed and weighted grooming networks. Spatial association networks were constructed using dyads that included the identity of the target individual and each animal within 3 m during the scan. We then extracted EC measures for non-adults from each network. Those with a higher EC score are connected to individuals who themselves are highly connected.

*iii. Neophilia*. We used estimates of neophilia calculated in a previous study where individuals were presented with novel food and tested on whether they would eat it (see Nord, 2021; Nord et al., 2022). In the previous study, we used Bayesian mixed effects modelling to estimate novel food neophilia. The estimates of the probability that individuals will eat a novel food served as an index for neophilia in our models.

*iv. Agonism*. Data on aggression were collected whenever observed. We recorded the identities of the aggressor, the victim, and the outcome from the perspective of the aggressor (win, loss, draw, or unknown; *N* = 50,924 occurrences). These data were used to calculate ranks for the entire group using the ‘EloRating’ package (Neumann & Kulik, 2020), allowing us to estimate a non-adult’s rank on the day of each observed intergroup encounter.

*v. Intergroup encounters*. We collected data on all observed intergroup encounters, which were scored when one or more members of one group directed their behaviour or moved towards individuals of another group after hearing or seeing members from a neighbouring group. We only included aggressive intergroup encounters in our analyses. An intergroup encounter was considered aggressive if one or more members behaved aggressively toward members of the other group. We collected data on all observed participants. In addition to recording group IDs and the number and IDs of participants, we scored the extent of participation, with the highest level observed for each participant being recorded. In ascending order, participants were ‘non-aggressive’ (at the immediate site of the IGC but did not otherwise participate), ‘stationary’ (offered facial or vocal threats), ‘active’ (lunged, charged at, or chased opponents), or ‘physical’ (slapped, grabbed or bit opponents). We were able to record the extent of participation for infants independent of their mother’s extent of participation. We also collected data on grooming interactions between bouts of conflict during IGC that were specific to the IGC. We recorded the IDs of all individuals that were grooming between bouts of conflict during intergroup encounters.

### 2.3. Statistical analyses

We constructed three Bayesian multilevel models using the ‘brms’ package (Bürkner, 2017) in R 4.4.2 (R-Core-Team, 2022) and a fourth in ‘rstan’ (Carpenter et al., 2017). All models were run on a data set including all non-adults (infants and juveniles), as well as on a subset containing only independent juveniles.

*i. Model 1*: What factors influenced the likelihood of participation (yes/no)? For each IGC, we entered participant age (days), sex, rank, neophilia score, spatial and grooming EC, whether the non-adult’s mother was a participant, the total number of participants from the non-adult’s group, the total number of participants from the opposing group, and the total group sizes as fixed effects. We specified an interaction between the number of participants from the non-adult’s group and the opposing group to test if a numerical advantage over the opposing group increases the likelihood of participation. We included non-adult ID, nested in its group (hereafter focal group) ID and opposing group ID as crossed random intercepts. We specified a Bernoulli distribution and ran the model with four chains and 2500 iterations.

*ii. Model 2*: What factors influenced the level of aggression shown by those non-adults that did participate? We used the same model structure and entered the same predictors as Model 1 but replaced maternal participation with the level of maternal aggression as a monotonic predictor, using the ‘mo’ function in brms to specify that maternal participation is ordinal (Bürkner, 2017). Both non-adult and maternal levels of aggression were ranked from least to most aggressive, using the maximum level of aggression recorded in the IGC data set (0 = non-participant, 1 = non-aggressive, 2 = stationary, 3 = active, 4 = physical). We specified an ordinal distribution and ran the model using 4 chains and 4000 iterations.

*iii. Model 3*: Was the receipt of grooming (yes/no) by non-adults at the time of the IGC related to their participation (yes/no)? We also included sex, age, rank, grooming and spatial EC, group size and IGC participant numbers as predictors. Non-adult ID, nested in focal group ID, was entered as a random intercept. We specified a Bernoulli distribution and ran the model with four chains and 3500 iterations.

*iv. Model 4*: What predicted whether non-adult participants were (i) groomed during the IGC and then, (ii) what predicted whether the groomer was their mother? To address this, we used the ‘rstan’ package to construct a nested double-hurdle structure model which is a model consisting of two hurdles. In our model, the first hurdle assesses whether each non-adult has been groomed. If they have been groomed, the second hurdle tests whether it was by their mother. Following the outcome of Model 3, we included sex, age and rank as predictors for both models. Non-adult ID, nested in maternal ID, nested in focal group ID was entered as a random intercept. We specified a Bernoulli distribution and ran the model using eight chains and 500 iterations.

All models were run with weakly informative priors (normal (0, 1)) and continuous variables were scaled and mean-centred. We used 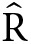
s to confirm convergence (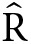 = 1.00) and evaluated model performance with the ‘pp_check()’ function from the ‘bayesplot’ package (Gabry & Mahr, 2017). Where appropriate, we assessed temporal autocorrelation in the residuals with brms’s ‘acf’ function. We set the credible intervals at 95% because of their interpretive familiarity and used these, backed by ‘probability of direction’ estimates from the ‘bayestestR’ package (Makowski, Ben-Shachar, & Lüdecke, 2019), to evaluate the size and precision of model outcomes (see Henzi et al., 2021). We calculated conditional and marginal *R*^2^ values for models 1–3 using the ‘bayes_R2’ function (Bürkner, 2017), recognizing that specifying an ordinal distribution in Model 2 requires caution in their interpretation. As *R*^2^ is an inefficient estimate of explained variance for Model 4, which predicts individual instances, we generated a receiver operating characteristic curve for each of the submodels and calculated the area under each curve (AUC).

All figures were created using the ‘ggplot2ʹ (Wickham, 2009) and ‘ggridges’ packages (Wilke, 2018).

## 3. Results

Non-adults participated in ∼79% of the 3350 observed IGCs. Most non-adult participation was non-aggressive (76.4%). They were, however, active participants in 13.4%, stationary participants in 8.9%, and physical participants in 1.4%. Mothers were co-participants in 41.5%. Participation was almost equal between the sexes (males: 50.4%; females: 49.6%). The average age of participants was 2.5 years, with the youngest being present as an inadvertent ventrally carried observer at 3 days of age, and the oldest being 5 years, which was the maximum age observed during the study period. The results remain consistent regardless of whether infants were included in the models (see Supplementary Figs. 1–4). The following results represent the findings for all non-adults.

**Figure 1.**
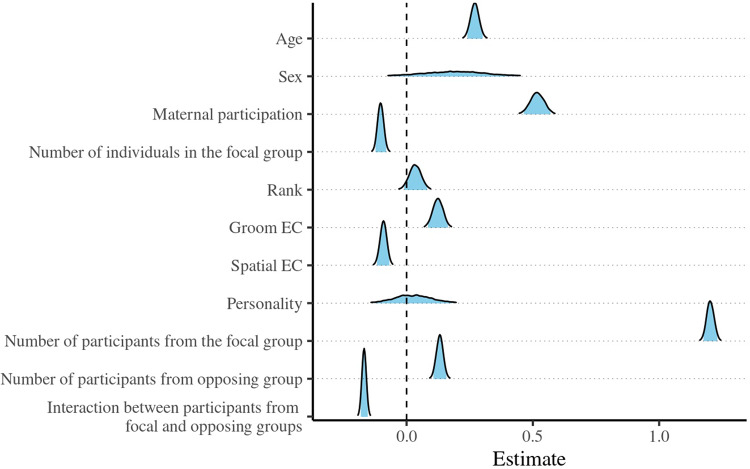
Posterior density estimates of the probability of participation (Y/N) in intergroup conflict in relation to age, sex (ref: female), maternal participation, the number of individuals in the troop, rank, grooming eigenvector centrality (EC), spatial EC, neophilia, the number of participants from the focal and opposing groups, together with their interaction. The blue fill is truncated to indicate the 95% credible intervals.

**Figure 2.**
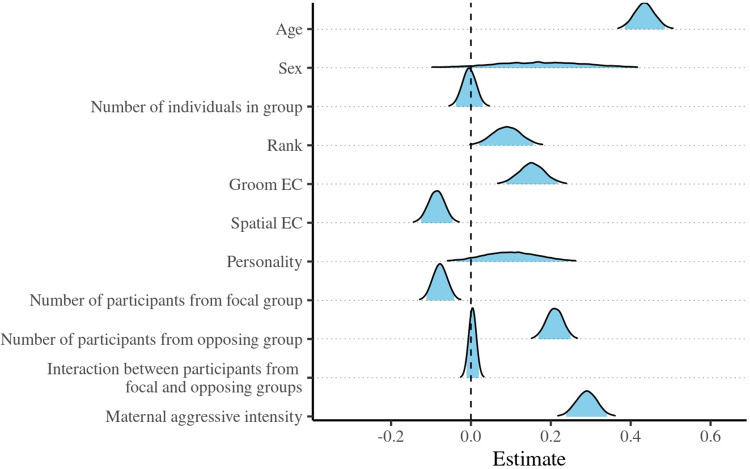
Posterior density estimates of changes in the level of aggressive intensity in relation to age, sex (ref: female), the number of individuals in the focal group, rank, grooming eigenvector centrality (EC), spatial eigenvector centrality, neophilia, the number of participants from the focal and opposing groups (and their interaction), and maternal aggressive intensity. The blue fill is truncated to indicate the 95% credible intervals.

**Figure 3.**
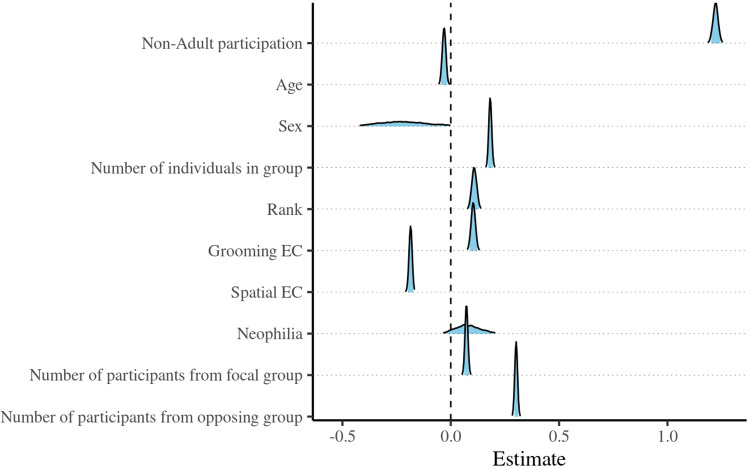
Posterior density estimates of the probability of grooming (Y/N) in relation to participation (Y/N), age, rank, neophilia, spatial eigenvector centrality, grooming eigenvector centrality, sex (ref: female), the number of individuals in the focal group, and the number of participants from the focal and opposing groups. The blue fill is truncated to indicate the 95% credible intervals.

**Figure 4.**
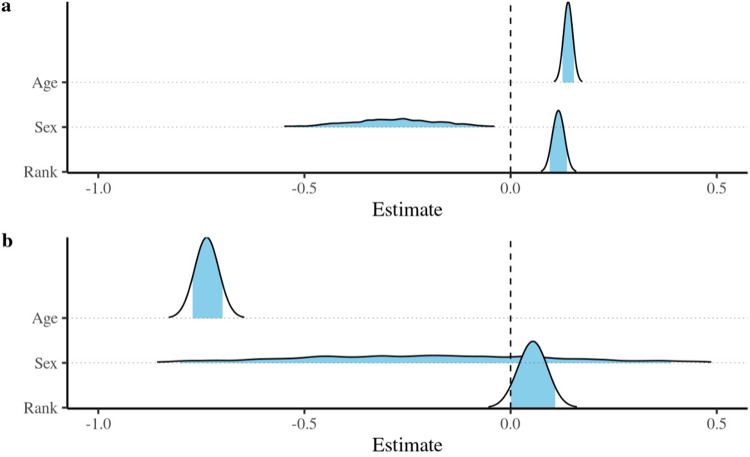
Posterior density estimates of the effects of sex, age and dominance rank on (a) the probability that non-adult participants would be groomed, and (b), if they were groomed, that it would be by their mothers. The blue fill is truncated to indicate the 95% credible intervals.

### 3.1. Predictors of non-adult participation in IGCs

We found that non-adults were more likely to participate as they got older, if their mother participated, and as the number of participants in their own group increased (Fig. 1, Supplementary Table 2). We also found small, precise positive effects for rank, grooming EC, and the number of participants in the opposing group. We found small, precise negative effects for focal group size, spatial EC, and the interaction between the number of participants in the contesting groups. Examination of the interaction indicates that it was driven largely by changes in the number of focal group participants (Supplementary Figure 5), with declining numbers reducing the likelihood of non-adult involvement. We found no meaningful effects for sex and our estimate of neophilia. The full model accounted for 28.5% of the variance (main effects: 24.2%).

### 3.2. Predictors of the level of aggression shown by non-adults during IGCs

Our second model (Fig. 2, Supplementary Table 3) showed that the likelihood of a non-adult being aggressive was positively associated with its age and the level of its mother’s involvement, with its level of aggression tracking that of its mother, and the effects being moderately strong and precise. There was reasonable evidence that neophilia was positively associated with levels of recorded aggression. We found the same contrast between grooming and spatial EC as in Model 1. Unlike the outcomes in Model 1, non-adults were less aggressive as focal group participant number increased, and more aggressive as the number of opposing group participants grew. We detected no interaction between these two variables. As in Model 1, we found no evidence of a sex difference. The full model accounted for 13.3% of the variance (main effects: 7.7%).

### 3.3. Grooming during the IGC and non-adult participation

Model 3 (Fig. 3, Supplementary Table 4) identified a strong and precise positive relationship between non-adult participation and the likelihood of being groomed, and smaller but precise positive relationships between grooming and focal group size, as well as participant number in both the focal and opposing groups. There was moderate evidence for a positive relationship between rank and grooming and the same opposing relationship for grooming and spatial EC. Interestingly, there was less precise but moderately strong evidence that non-adult males were less likely to be groomed during IGCs. We detected little evidence of effects for age and neophilia. The full model accounted for 12.2% of the variance (main effects: 9.3%).

### 3.4. Grooming of non-adult participants and the role of the mother

Model 4 (Fig. 4, Supplementary Tables 5 and 6) indicates non-adult participants were more likely to be groomed if they were older, whereas there was strong, moderately precise evidence that this was more likely to be by their mother if they were younger (Fig. 4a). There was a small, precise effect for rank, with higher-ranking participants more likely to be groomed, although there was little evidence that mothers differentiated in this way. Similarly, although there was moderate, imprecise evidence that male participants were less likely to be groomed than females, there was little suggestion that mothers discriminated by offspring sex. AUC values for the first hurdle model were 0.62 and 0.54 for the full model and main effects, respectively; those for the second hurdle model were 0.77 and 0.63.

## 4. Discussion

Our results indicate that the likelihood and intensity of non-adult involvement in IGCs were associated with a suite of individual, situational and social factors. Unsurprisingly, older (and therefore larger, more experienced) non-adults were increasingly likely both to participate and to do so with greater intensity. By the same token, neither males, despite greater weight-for-age (Jarrett et al., 2020), nor females, despite philopatric commitments to territorial defence (Cords, 2007; Heinsohn et al., 1996), were more invested in participation. Although rank predicted participation, it had no effect on levels of aggression. In contrast, neophilia, as our index of boldness, although not predictive of participation itself, was associated with higher levels of aggression. The effect is small but in line with general expectation (Briffa, Sneddon, & Wilson, 2015) and, alongside evidence of temperamental consistency in the species (Blaszczyk, 2018), may provide evidence of the early emergence of individuals that are ‘key’ to success in intergroup contests (Glowacki & McDermott, 2022).

Non-adults were also clearly sensitive to numerical asymmetries, being slightly less likely to participate if the overall size of their group was larger than that of their opponents; a finding in line with both theoretical (Olson, 1965) and empirical (Crofoot & Gilby, 2012; Mirville et al., 2018) expectations of increased free-riding in larger groups, at least by adult participants. They were, however, much more likely to participate themselves as the number of active participants – especially those from their own groups – increased (Model 1; Supplementary Fig. 1). Rather than shying away from engagement, therefore, their engagement was proximally promoted by numerical advantage, suggesting that this buffered them from the increased risks they faced by virtue of their size and inexperience. Once committed to an IGC, and in line with this, the intensity of their aggression was negatively associated with the number of focal group participants, but increased with the number of participants in the opposing group (Model 2). In this regard, their behaviour matched theoretical expectation, with greater commitment in the face of increased threat.

Sociospatial integration measures were meaningful predictors in Models 1 and 2 but in different directions, with increased grooming EC being positively – and spatial EC negatively – associated with both participation and aggressive intensity. This apparent contradiction may perhaps be accounted for by the fact that grooming and spatial networks are generally dissociated in our population (Henzi, Forshaw, Boner, Barrett, & Lusseau, 2013). More specifically, non-adult spatial ties are to other non-adults, whereas grooming ties are strongly centred on their mothers (Vilette et al., 2023). Increased spatial integration, therefore, binds non-adults to non-adult group members who are intrinsically to participate and to do so with lower intensity (Cords, 2007), whereas social integration runs through the mother.

By far the strongest positive predictor of non-adult grooming at the time of an IGC event was participation, followed by rank, grooming centrality, and situational conditions (group size, participant numbers) that likely reflected the general tenor and intensity of the IGC. There was no effect of age, despite the increasing likelihood of engagement. At the same time, despite the absence of sex differences in participation and aggression, and unlike the general pattern of grooming (Jarrett et al., 2018), females were more likely to be groomed during IGCs regardless of participation. Narrowing the analysis to participants indicated that grooming with non-adult participants was more likely if they were older, higher-ranking, or female, but that mothers themselves preferentially targeted only younger, more vulnerable offspring.

In concert, and reflecting the value of grooming as a ‘carrot’ (Arseneau-Robar et al., 2016), these outcomes are consistent with the differential reinforcement of attributes – higher rank, social connectedness and philopatry – likely to be important in the future, and, as indexed by age, current IGC effort. The role of the mother in the induction of offspring should be evaluated in this context. Although our non-adults do not inherit maternal grooming networks, they do track them, and maternal self-similarity and integration are predictive of non-adult integration (Jarrett et al., 2018). This, along with the finding that maternal involvement and levels of aggression were the best social predictors of non-adult patterns of participation, suggests that mothers serve both as direct and, through their close adult associates, indirect drivers of differential non-adult engagement in IGCs. The similar outcomes of the analyses restricted to independent juveniles, which we do not report here, confirm that the observed maternal effects are not confounded with infant dependency.

Our findings support the idea that IGC participation can be promoted and potentially inculcated in young non-human animals despite the absence of formal cultural rules and linguistic social practices. There are, as always, reasons to urge caution in the direct extrapolation of these outcomes. First, IGC occurs at very high frequency in our population, and non-adults are exposed both regularly and frequently to aggression of this kind. They consequently have more opportunities simply to acquire the ‘habit’ or ‘norm’ of participation more readily than in other populations. Then, we cannot exclude the possibility that vervets possess some intrinsic potential to behave aggressively toward strange conspecifics, such that they are not being inducted into this behaviour via social processes, but simply maturing into it. This seems unlikely, given the patterning of our findings, but cannot be ruled out. Equally, it is not clear that IGC participation represents any kind of genuine prosocial behaviour, in the sense that animals possess ‘other regarding’ preferences and act to generate a public good, and that animals can be free-riders under come conditions. That is, even if we can accept that non-adults are inducted to engage in IGC via social processes, we should still remain sufficiently skeptical, at present, as to whether these findings offer a useful and productive analogue for understanding the evolution of prosocial behaviour in our own species.

Assuming that our findings, at the very least, support the existence of social induction, we still need to characterize exactly what it is that non-adults are learning through development: is it both to respond aggressively to strangers, and to participate in IGC in risk-sensitive ways? Or are they discovering how to moderate an inherently aggressive response to strangers via attunement to the behaviour of their mothers and others, and responding to the contingencies of aggressive action and grooming rewards? Given previous findings on the development of anti-predator vocal communication (Dubreuil, Barrett, Henzi, Notman, & Pavelka, 2023), the latter is more likely, but it remains an open question.

If we accept that induction into (potentially pro-) social behavior occurs, then we can consider some intriguing corollaries. For example, Vygotsky (1987) argued that human children participate in social practices well before they have the capacity to understand them, and that it is only through this participation and – crucially – being treated by adults as though they already have the necessary comprehension, that they are able to develop an understanding of the situations in which they find themselves. Obviously, as stressed in the introduction, this is achieved through exposure to linguistic and highly structured sociocultural environments. Without drawing false comparisons, there is, however, some resemblance to the vervets here, in that much of children’s early cultural participation involves highly interactive, physically embodied activities – to put it colloquially, they simply take part, pitch in and have a go – and they respond, resist and react to the equally embodied social feedback they receive from those around them.

It is in this more limited sense that Vygotsky’s ideas resonate with our finding that one of the best predictors of any kind of non-adult participation in our study was their mother’s own participation – non-adult vervets also seem to be thrown into a world in which they first participate, and then gradually refine their responses with greater experience. Further, it suggests that a productive route for comparative analyses would be to build from the bottom-up, paying more attention to the kinds of embodied, sensorimotor socially interactive patterns that are shared by humans and non-human primates alike, and how these might form a scaffold for the kinds of highly elaborated sociocultural practices that facilitate the equally elaborate forms of coordination and cooperation that are so characteristic of human groups, both past and present (Barrett, Henzi, & Barton, 2022; see also Graziano, 2017).

## Supporting information

Clarke et al. supplementary material 1 Clarke et al. supplementary material

Clarke et al. supplementary material 2 Clarke et al. supplementary material

Clarke et al. supplementary material 3 Clarke et al. supplementary material

Clarke et al. supplementary material 4 Clarke et al. supplementary material

Clarke et al. supplementary material 5 Clarke et al. supplementary material

Clarke et al. supplementary material 6 Clarke et al. supplementary material

Clarke et al. supplementary material 7 Clarke et al. supplementary material

Clarke et al. supplementary material 8 Clarke et al. supplementary material

Clarke et al. supplementary material 9 Clarke et al. supplementary material

Clarke et al. supplementary material 10 Clarke et al. supplementary material

Clarke et al. supplementary material 11 Clarke et al. supplementary material

## Data Availability

The code and the data used are available online at https://github.com/MadisonClarke/Social-Induction/tree/V1.1.

## Supplementary material

The supplementary material for this article can be found at https://doi.org/10.1017/ehs.2025.7.
