# Supplementary figures and images for: Social induction and the developmental trajectory of participation in intergroup conflict by vervet monkeys
Source: Evol Hum Sci. 2025 Mar 13;7:e9. doi: 10.1017/ehs.2025.7 (PMC11949634; doi:10.1017/ehs.2025.7)

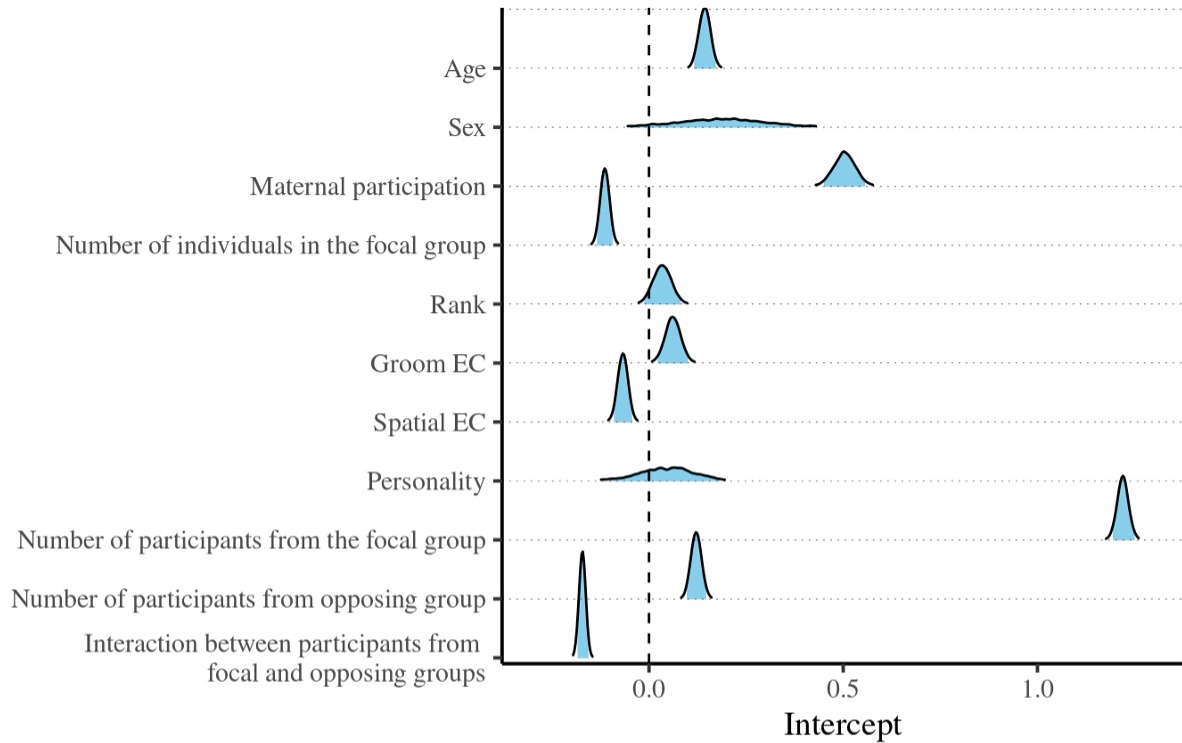

Supplement: Clarke et al. supplementary material 1 — Clarke et al. supplementary material [file S2513843X25000076sup001.pdf]

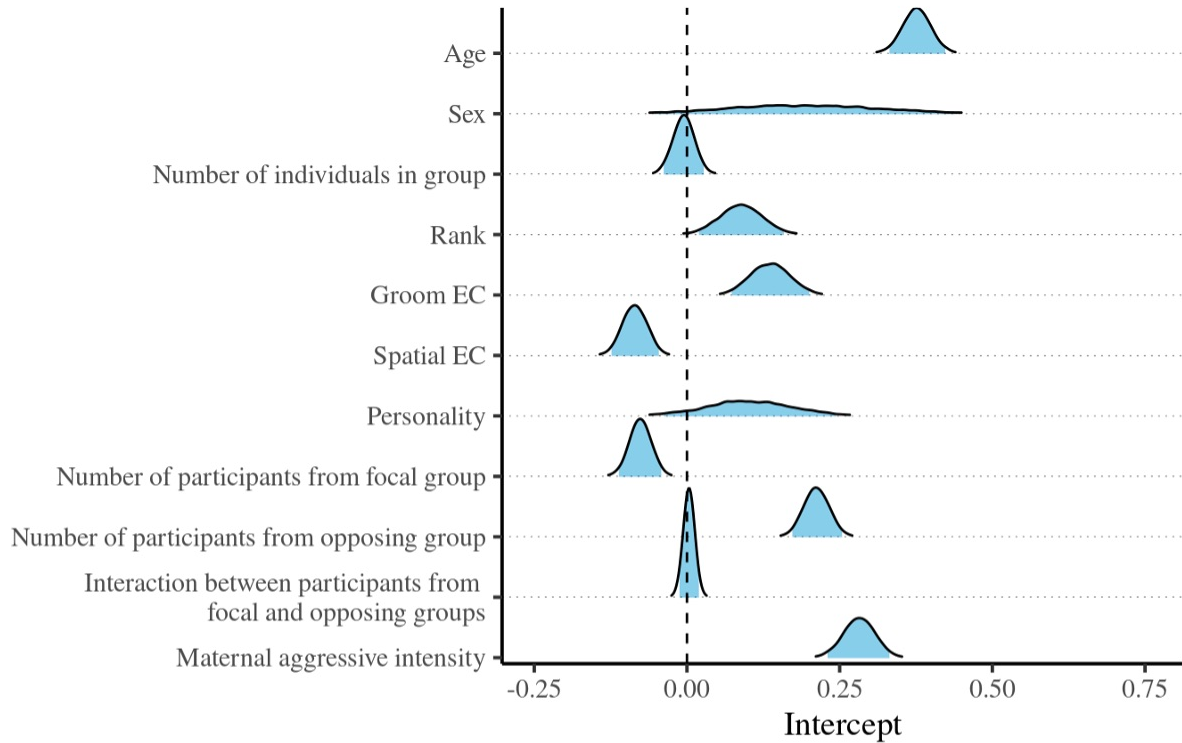

Supplement: Clarke et al. supplementary material 2 — Clarke et al. supplementary material [file S2513843X25000076sup002.pdf]

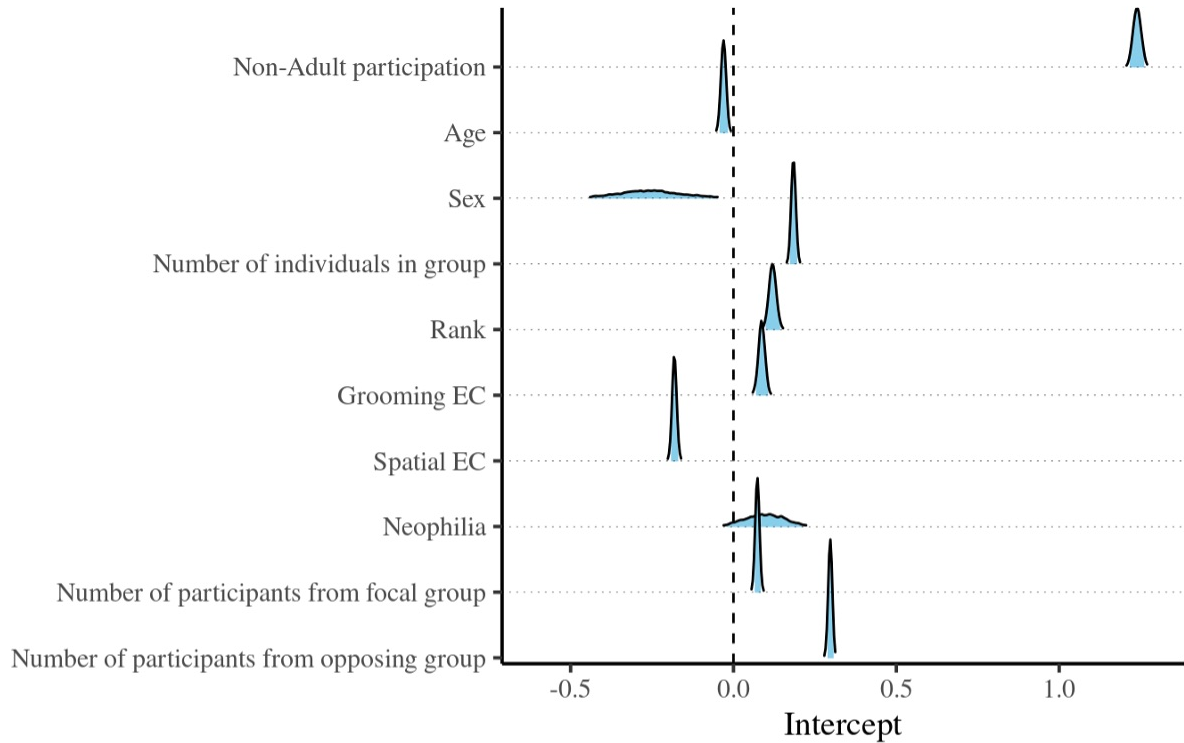

Supplement: Clarke et al. supplementary material 3 — Clarke et al. supplementary material [file S2513843X25000076sup003.pdf]

**a**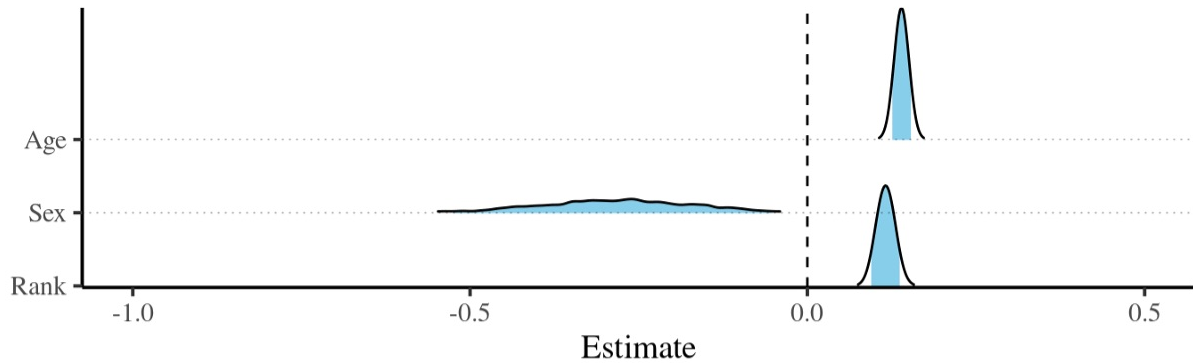**b**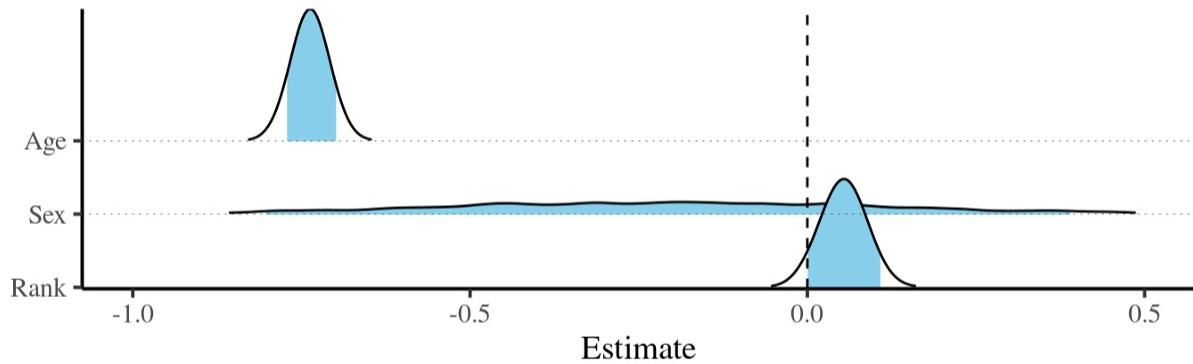

Supplement: Clarke et al. supplementary material 4 — Clarke et al. supplementary material [file S2513843X25000076sup004.pdf]

Participants in opposing group

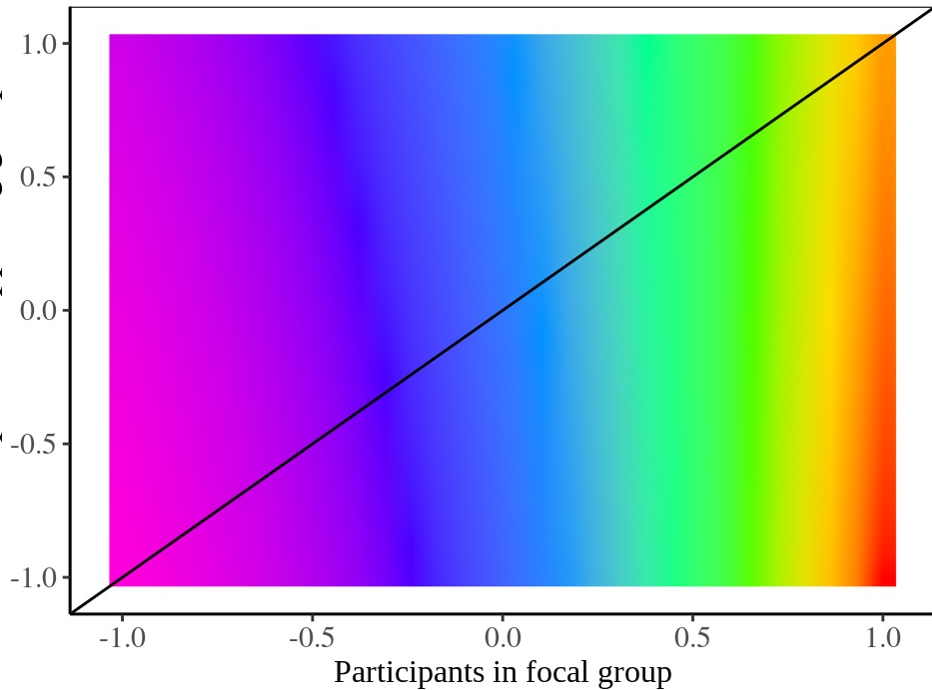

Supplement: Clarke et al. supplementary material 5 — Clarke et al. supplementary material [file S2513843X25000076sup005.pdf]
