# Supplementary material for: Social induction and the developmental trajectory of participation in intergroup conflict by vervet monkeys
Source: Evol Hum Sci. 2025 Mar 13;7:e9. doi: 10.1017/ehs.2025.7 (PMC11949634; doi:10.1017/ehs.2025.7)
Supplement: Clarke et al. supplementary material 6 — Clarke et al. supplementary material [file S2513843X25000076sup006.pdf]

Supplementary Table 1. *Troop composition for study period (2014-2018).*

Year	Troop	Age and Sex	Count
2014	PT	AF	11
	PT	AM	6
	PT	JF	5
	PT	JM	4
	RST	AF	18
	RST	AM	26
	RST	JF	6
	RST	JM	6
	RBM	AF	12
	RBM	AM	22
	RBM	JF	5
	RBM	JM	5
2015	PT	AF	10
	PT	AM	7
	PT	JF	9
	PT	JM	8
	RST	AF	18
	RST	AM	25
	RST	JF	10
	RST	JM	12
	RBM	AF	12

	RBM	AM	15
	RBM	JF	11
	RBM	JM	11
2016	PT	AF	10
	PT	AM	12
	PT	JF	9
	PT	JM	15
	RST	AF	17
	RST	AM	23
	RST	JF	14
	RST	JM	15
	RBM	AF	10
	RBM	AM	15
	RBM	JF	15
	RBM	JM	13
2017	PT	AF	9
	PT	AM	12
	PT	JF	9
	PT	JM	15
	RST	AF	20
	RST	AM	21
	RST	JF	14
	RST	JM	12

	RBM	AF	8
	RBM	AM	13
	RBM	JF	15
	RBM	JM	13
2018	PT	AF	11
	PT	AM	9
	PT	JF	8
	PT	JM	13
	RST	AF	19
	RST	AM	24
	RST	JF	5
	RST	JM	9
	RBM	AF	9
	RBM	AM	11
	RBM	JF	13
	RBM	JM	13

AF: Adult Female, AM: Adult Male, JF: Juvenile Female, JM: Juvenile Male.
