# Supplementary material for: Social induction and the developmental trajectory of participation in intergroup conflict by vervet monkeys
Source: Evol Hum Sci. 2025 Mar 13;7:e9. doi: 10.1017/ehs.2025.7 (PMC11949634; doi:10.1017/ehs.2025.7)
Supplement: Clarke et al. supplementary material 8 — Clarke et al. supplementary material [file S2513843X25000076sup008.pdf]

Supplementary Table 3. *Posterior estimates of changes in the level of aggressive intensity in relation to age, sex (Ref: Female), the number of individuals in the focal group, rank, grooming eigenvector centrality (EC), spatial eigenvector centrality, neophilia, the number of participants from the focal and opposing groups (and their interaction), and maternal aggressive intensity.*

	**β**	**SE**	**Lower-95% CI**	**Upper-95% CI**	**ESS**	**PD (%)**
Intercept [1]	1.72	0.18	1.33	2.05	3696.85	100
Intercept [2]	0.31	0.18	-0.09	0.63	3632.83	95.25
Intercept [3]	3.26	0.19	2.85	3.61	3841.40	100
Age	0.44	0.03	0.39	0.48	10710.29	100
Sex (Ref: Female)	0.20	0.13	-0.06	0.45	6411.63	93.8
Number of individuals in the focal troop	-0.00	0.02	-0.04	0.03	13115.51	58.86
Rank	0.00	0.03	-0.07	0.07	11053.30	50.34
Grooming EC	0.16	0.03	0.09	0.22	12190.95	100
Spatial EC	-0.09	0.02	-0.13	-0.05	14290.55	100
Neophilia	0.15	0.06	0.03	0.28	6475.63	99.11
Number of participants from the focal group	-0.08	0.02	-0.11	-0.04	12966.23	100
Number of participants from the opposing group	0.21	0.02	0.17	0.25	12225.39	100
Interaction between the number of participants from the focal and opposing groups	0.00	0.01	-0.01	0.02	11522.66	71.09
Maternal aggressive intensity	0.29	0.03	0.24	0.34	9363.26	100

ID, nested in troop, and opposing troop identity were entered as crossed random intercepts. β: slope of the predictor; SE: standard error of the estimate of β; CI: credible interval; ESS: effective sample size; PD: probability of direction. $R^2_{marginal}$=0.08. $R^2_{conditional}$=0.13. N=16,918.
