# Supplementary material for: Social induction and the developmental trajectory of participation in intergroup conflict by vervet monkeys
Source: Evol Hum Sci. 2025 Mar 13;7:e9. doi: 10.1017/ehs.2025.7 (PMC11949634; doi:10.1017/ehs.2025.7)
Supplement: Clarke et al. supplementary material 9 — Clarke et al. supplementary material [file S2513843X25000076sup009.pdf]

Supplementary Table 4. *Posterior estimates of the probability of grooming (Y/N) in relation to participation (Y/N), age, rank, neophilia, spatial eigenvector centrality, grooming eigenvector centrality, sex (Ref: Female), the number of individuals in the focal group, the number of participants from the focal and opposing groups.*

	**β**	**SE**	**Lower-95% CI**	**Upper-95% CI**	**ESS**	**PD (%)**
Intercept	-2.58	0.08	-2.74	-2.41	2673.28	100
Non-adult participation	1.40	0.03	1.33	1.46	8861.34	100
Age	0.02	0.02	-0.02	0.06	7203.83	84.51
Rank	0.07	0.03	0.01	0.13	6160.57	99.47
Neophilia	0.04	0.06	-0.08	0.16	2521.25	76.5
Spatial eigenvector centrality	-0.17	0.02	-0.20	-0.14	8876.59	100
Grooming eigenvector centrality	0.09	0.03	0.03	0.14	6655.81	99.96
Sex (Ref: Female)	-0.31	0.11	-0.53	-0.09	2428.51	99.71
Number of individuals in the group	0.12	0.02	0.09	0.15	7670.87	100
Number of participants from the focal group	0.23	0.02	0.19	0.26	7672.86	100
Number of participants from the opposing group	0.29	0.016	0.26	0.32	7820.93	100

ID and troop were entered as nested random intercept. β: slope of the predictor; SE: standard error of the estimate of β; CI: credible interval; ESS: effective sample size; PD: probability of direction. $R^2_{marginal}$=0.09. $R^2_{conditional}$=0.12. N=57,138.
