# Supplementary material for: Social induction and the developmental trajectory of participation in intergroup conflict by vervet monkeys
Source: Evol Hum Sci. 2025 Mar 13;7:e9. doi: 10.1017/ehs.2025.7 (PMC11949634; doi:10.1017/ehs.2025.7)
Supplement: Clarke et al. supplementary material 10 — Clarke et al. supplementary material [file S2513843X25000076sup010.pdf]

Supplementary Table 5 *Posterior estimates of the probability of grooming (Y/N) in relation to age, rank, and sex (Ref: Female).*

	**β**	**SE**	**Lower-95% CI**	**Upper-95% CI**	**ESS**	**PD (%)**
Intercept	-0.83	0.01	-1.31	-0.27	615	99
Age	0.14	0.00	0.13	0.15	2267	100
Rank	0.12	0.00	0.09	0.14	4126	100
Sex	-0.28	0.00	-0.53	-0.05	1479	99.05

ID, mother ID, and troop were entered as nested random intercept. β: slope of the predictor; SE: standard error of the estimate of β; CI: credible interval; ESS: effective sample size; PD: probability of direction. AUC= 0.62 (full model) 0.54 (main effects).
