# Supplementary material for: Social induction and the developmental trajectory of participation in intergroup conflict by vervet monkeys
Source: Evol Hum Sci. 2025 Mar 13;7:e9. doi: 10.1017/ehs.2025.7 (PMC11949634; doi:10.1017/ehs.2025.7)
Supplement: Clarke et al. supplementary material 11 — Clarke et al. supplementary material [file S2513843X25000076sup011.pdf]

Supplementary Table 6 *Posterior estimates of the probability of mother grooming (Y/N) in relation to age, rank, and sex (Ref: Female).*

	**β**	**SE**	**Lower-95% CI**	**Upper-95% CI**	**ESS**	**PD (%)**
Intercept	-1.22	0.02	-2.37	0.14	1417	96.35
Age	-0.74	0.00	-0.77	-0.70	3631	100
Rank	0.05	0.00	0.00	0.11	3375	97.4
Sex	-0.22	0.01	-0.93	0.44	1741	72.55

ID, mother ID, and troop were entered as nested random intercept. β: slope of the predictor; SE: standard error of the estimate of β; CI: credible interval; ESS: effective sample size; PD: probability of direction. AUC= 0.77 (full model) 0.63 (main effects).
